# In depth characterisation of the biomolecular coronas of polymer coated inorganic nanoparticles with differential centrifugal sedimentation

**DOI:** 10.1038/s41598-021-84029-8

**Published:** 2021-03-19

**Authors:** André Perez-Potti, Hender Lopez, Beatriz Pelaz, Abuelmagd Abdelmonem, Mahmoud G. Soliman, Ingmar Schoen, Philip M. Kelly, Kenneth A. Dawson, Wolfgang J. Parak, Zeljka Krpetic, Marco P. Monopoli

**Affiliations:** 1 Centre for Bionano Interactions, University College Dublin, Dublin, Ireland; 2 Department of Medicine Huddinge, Center for Infectious Medicine, Karolinska Institutet, Stockholm, Sweden; 3 School of Physics and Optometric & Clinical Sciences, Technological University Dublin, City Campus, Kevin Street, Dublin 8, Ireland; 4 Fachbereich Physik, CHyN, University of Hamburg, Hamburg, Germany; 5 Centro Singular de Investigación en Química Biolóxica e Materiais Moleculares (CiQUS), Universidade de Santiago de Compostela, 15782 Santiago, Spain; 6 Departamento de Química Inorgánica, Grupo de Física de Coloides y Polímeros, Universidade de Santiago de Compostela, 15782 Santiago, Spain; 7 Food Technology Research Institute, Agricultural Research Center, Cairo, Egypt; 8 Institut für Physikalische Chemie und Elektrochemie, Leibniz Universität Hannover, Hannover, Germany; 9 Chemistry Department, RCSI (Royal College of Surgeons in Ireland), 123 St Stephen Green, Dublin 2, Ireland; 10 Physics Department, Faculty of Science, Al-Azhar University, Cairo, Egypt; 11 Biomedical Research Centre, School of Science Engineering and Environment, University of Salford, Salford, M5 4WT UK; 12 School of Pharmacy and Biomolecular Sciences, Royal College of Surgeons in Ireland, 123 St Stephen Green, Dublin 2, Ireland

**Keywords:** Chemistry, Nanoscience and technology

## Abstract

Advances in nanofabrication methods have enabled the tailoring of new strategies towards the controlled production of nanoparticles with attractive applications in healthcare. In many cases, their characterisation remains a big challenge, particularly for small-sized functional nanoparticles of 5 nm diameter or smaller, where current particle sizing techniques struggle to provide the required sensitivity and accuracy. There is a clear need for the development of new reliable characterisation approaches for the physico-chemical characterisation of nanoparticles with significant accuracy, particularly for the analysis of the particles in the presence of complex biological fluids. Herein, we show that the Differential Centrifugal Sedimentation can be utilised as a high-precision tool for the reliable characterisation of functional nanoparticles of different materials. We report a method to correlate the sedimentation shift with the polymer and biomolecule adsorption on the nanoparticle surface, validating the developed core–shell model. We also highlight its limit when measuring nanoparticles of smaller size and the need to use several complementary methods when characterising nanoparticle corona complexes.

## Introduction

Progressive advancements in nanofabrication approaches have led to the development of nanoparticles (NP) of various core materials, size and shape that have found applications in a broad range of sectors, from life science, and electronics to energy harvesting. The NP used as a platform in healthcare has also raised significant interest due to the development of NP-based medical devices for biomolecular sensing, diagnostics, and also nanovectors for drug delivery in cancer treatment^1–3^.

Despite these numerous applications, these advanced functional nanomaterials require precise control of the surface chemistry to enable targeting, sensing, and drug delivery. With the addition of drugs or other functional moieties to the NP ligand shells, the characterisation of the functionalised particles can become complex, requiring multiple analytical techniques to provide a full picture and achieve a robust characterisation to enable use in clinical trials and nanomedicine^4^.

NPs are often subjected to a range of controlled stages at the manufacturing process, spanning from production to purification, through to characterisation and integration in consumer products. Where applicable, nanomaterials must comply with required regulatory legislation and standards^5,6^.

Due to their unique size and surface properties, NPs have the potential to engage with cellular machinery with high affinity, precision and specificity opening up the potential for tailoring their biodistribution; though in vivo targeting remains a major issue^7,8^. In many in vitro and in vivo scenarios, NPs strongly interact with proteins and other biomolecules readily available in biological fluids to which they are exposed to, often forming the so called ‘biomolecular corona’^9–11^. From many reports, it is clear that the biomolecular corona may impact the identity and alter the behaviour of the NPs depending on its composition and topological organisation, leading to the presence of specific motifs i.e. epitopes of the adsorbed biomolecules, prone to be recognised with high affinity and specificity by the cell receptors, and therefore often steering the interactions and uptake routes^12–18^.

In addition to the formation of altered biological identity, the biomolecular corona may also influence the physico-chemical properties of the NPs, e.g. dispersion, hydrodynamic diameter, surface charge, colloidal stability and degradation, which can impact their pharmacokinetic properties and biodistribution^19–23^.

NPs of 5 nm diameter or smaller are gaining attention in the field of nanomedicine^24^. In contrast to the larger ones, these nanomaterials offer improved tissue penetration, a reduced accumulation in the liver and more efficient renal clearance^25^, which renders them particularly attractive for reducing the non-specific accumulation and off-target effects.

Within this context, metallic NPs, such as gold (AuNPs) or semiconductor quantum dots (QDs) have become a popular choice. They can be easily synthesised in a size-controlled manner, within the desirable size range, with good size monodispersity, high batch reproducibility and highly tuneable surface chemistry which allows for a tailored design^21^. They are generally synthesised in an organic media using a biocompatible polymers, e.g. polyethylene glycol or chitosan amongst others, which is grafted onto the NP surface to ensure colloidal stability during the phase transfer into the aqueous media. The addition of this layer inevitably leads to a small increase in their diameter and changes in the overall density. This layer is however essential to ensure the colloidal stability even after the exposure to physiological solutions^1,26^. As such, characterisation of the newly functionalised particles is of utmost importance to ensure successful surface functionalisation and monitor particle stability over time under a variety of dispersion conditions^4^.

Despite the broad interest, current limitations in the use of small NPs were reported due to the challenges associated with their physico-chemical characterisation, e.g. size distribution measurements, in particular after bioconjugation^27^, commonly characterised using commercially available techniques such are Dynamic Light Scattering (DLS) and Nanoparticle Tracking Analysis (NTA) which struggle to analyse such challenging objects due to their detection limit and other issues^28,29^. Other techniques, such as Fluorescence Correlation Spectroscopy (FCS) or agarose gel electrophoresis (AGE) require particular particle properties (e.g. fluorescence) and are not broadly applicable to all NP types^4^. Recent studies reported the use of analytical or ultra-centrifugation as an alternative method to characterise the particle size distribution of a wide range of nanomaterials^30–32^. The establishment of a characterisation method to reliably study the NP characterisation after exposure in complex biological media (e.g. human plasma or serum) or in situ is still required so that the reproducibility and confidence in the production of such materials is achieved.

In this manuscript, we developed a series of inorganic NPs of *ca*. 5 nm core diameters, i.e. with a core of gold, CdSn/ZnS quantum dots, silver, or iron platinum functionalised with a commonly used versatile and biocompatible polymer, poly-(isobutylene-alt-maleic anhydride)-graft-dodecyl (PMA)^4^, along with a series of AuNPs of three different sizes but same surface coating, and we used Differential Centrifugal Sedimentation (DCS), an emerging technique of growing popularity for  the NP characterisation.

The DCS is a simple technique that measures the size distribution based on the sedimentation time of a particle through a sucrose density gradient placed in a spinning disc. Under these conditions, the particles will separate according to their size and density. During a typical experimental run, the instrument measures the time that elapses from the moment in which the NPs enter the gradient and reach the detector. From this time, the NP diameter is calculated using modified Stoke’s law. However, when a NP is coated with a polymer or with a biomolecule and its resulting density and size is changed, the NP sedimentation size will be altered, and the size mesured by the DCS is not correct. Therefore, the identification of the “true” NP size from the "apparent" size obtained in DCS measurements requires using a simple core-shell model that takes into account the new NP density. DCS allows a high-resolution separation and detection of a small percentage of particle populations within the polydisperse colloidal samples analysed that are not detectable by other techniques, and can also provide useful information on the surface functionalisation with stabilising ligands and overall colloidal stability after biomolecular interaction^13,33,34^.

Additionally, while on the manufacturer information the DCS is claimed to be capable of measuring the NP size in the range between 3 nm and 60 μm, most studies to date are focused on NP with diameters of 20 nm or larger and with high density. For consistency and validation of our results, we compared the particle size measurements obtained from DCS measurements of the 5 nm core particles with a wider set of particles of the same core material but of larger particle diameter, i.e. 25 and 50 nm. Finally, we characterised these nanomaterials after exposure to blood plasma, a biological fluid of particular interest used in nanomedicine and nanosafety studies.

**Scheme 1:**
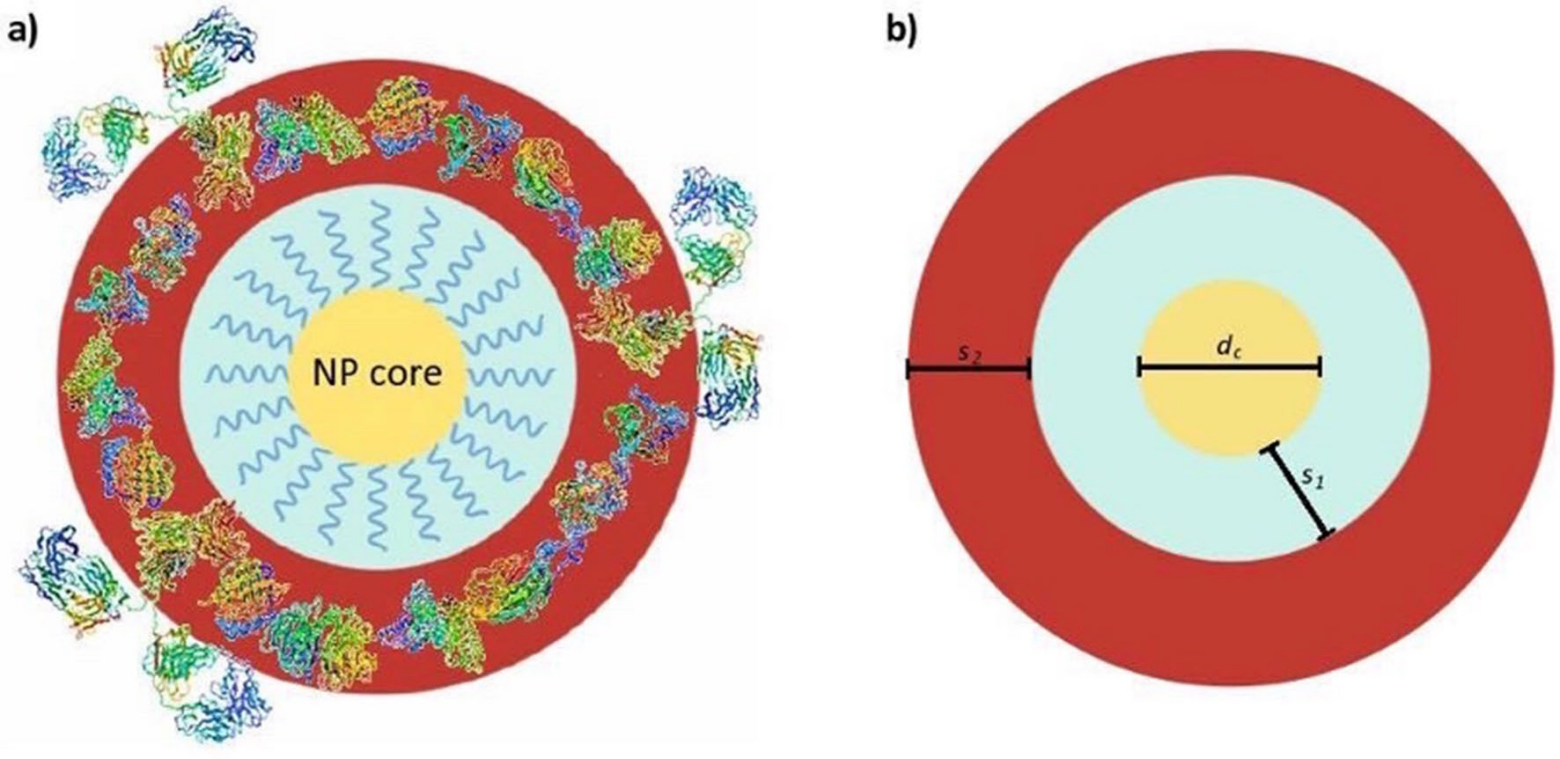
(**a**) Schematic showing the proposed complex core–shell model used for the analysis of the particle diameter by Differential Centrifugal Sedimentation (DCS) for polymer functionalised inorganic nanomaterial forming the NP-biomolecular corona complexes containing several layers of different intrinsic thickness and density. (**b**) *d*_*c*_ nanoparticle core diameter; *s*_*1*_*:* shell thickness of the polymer coating; *s*_*2*_: protein corona shell thickness. Scheme drawn with Microsoft PowerPoint and the proteins structures were adapted from the Protein Data Bank (http://www.pdb.org).

Herein, we highlight the main challenges associated with sizing polymer functionalised inorganic NP, using a core–shell sedimentation model (Scheme 1) and we show the differences in the sedimentation behaviour not commonly occurring with particles of larger diameters.

## Results and discussion

### Determination of particle size distribution of polymer-coated NPs via DCS technique

**Figure 1:**
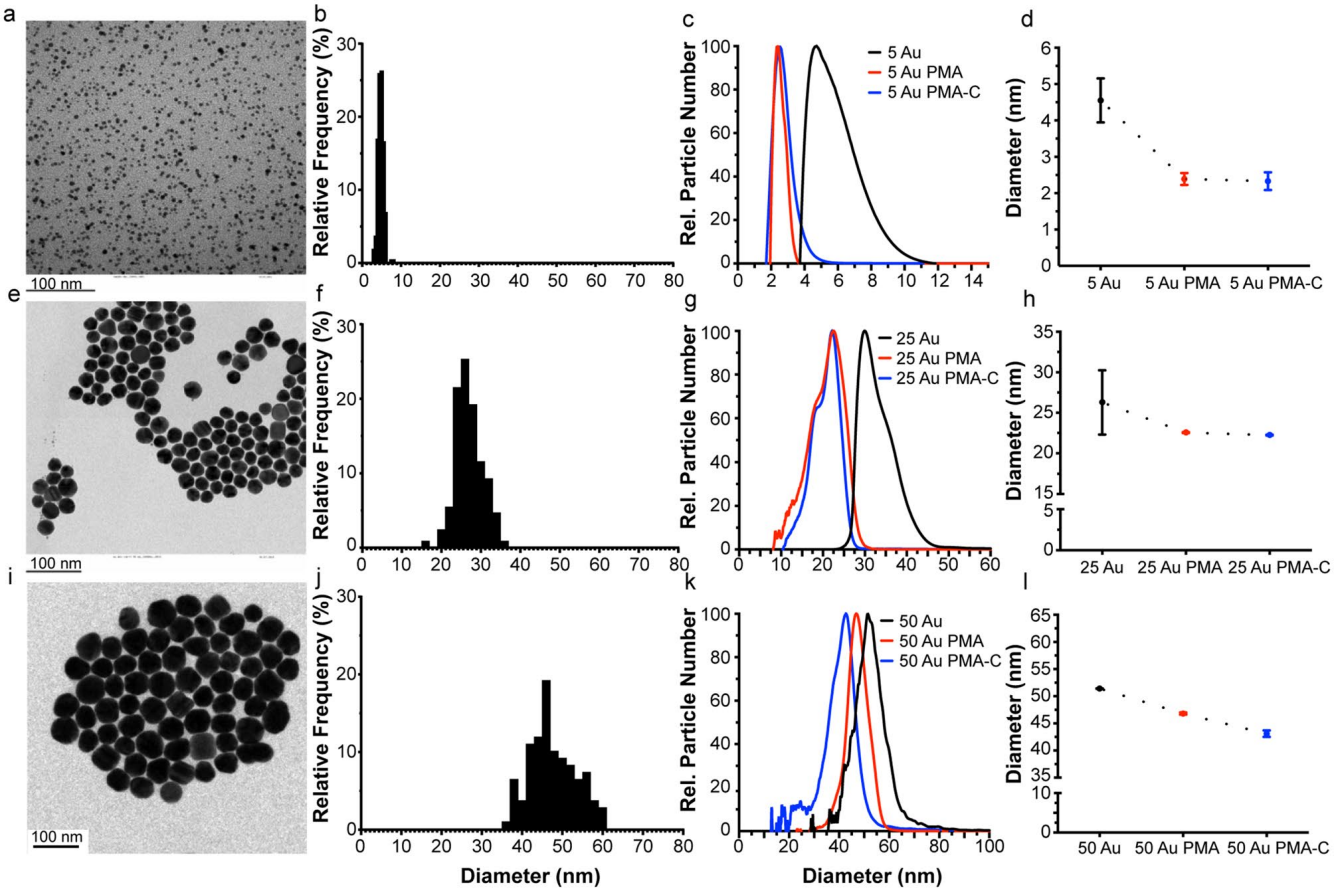
Characterisation of PMA coated AuNPs of different sizes by TEM and DCS. Measurements for 5, 25 and 50 AuNPs are shown respectively in the top (**a**–**d**), middle (**e**–**h**) and bottom (**i**–**l**) panels. TEM measurements were performed on the polymer-coated NPs. Representative micrographs for each of the different sizes (**a**,**e**,**i**) and the resulting histogram counts (**b**,**f**,**j**) are provided with average core diameters of 4.8 ± 0.7, 26.9 ± 3.4 and 47.6 ± 5.7 nm respectively. DCS measurements were performed for the three relevant NP suspensions, i.e. AuNPs in the organic solvent (toluene) prior to PMA coating (black line), the Au-PMA polymer-coated NPs after water transfer (red line) and Au-PMA-Corona complexes in situ measured after corona formation (blue line). Representative DCS distributions are provided for each of the NP sizes (**c**,**g**,**k**) along with the detailed average and standard deviations across independent replicates (**d**,**h**,**l**). Particle diameters obtained from DCS measurements assumed a particle density of 19.3 g cm^-3^.

Spherical gold NPs of 5 nm diameter were synthesised following Brust-Shiffrin method^35^. While the NP synthesis was carried out in toluene, we applied a polymeric surface grafting using a hydrophilic layer of poly-(isobutylene-alt-maleic anhydride)-graft-dodecyl (PMA), necessary to facilitate the phase transfer from toluene into the water, that also ensures particle stability in aqueous media^36,37^. Particles were sized using Transmission Electron Microscopy (TEM) showing an average core diameter of 4.8 ± 0.7 nm while the organic surface coating was not visible with this technique (Fig. 1a,b, Table 1)^4^. The particle stability was assessed with DCS and comparable particle size distribution of the TEM measurements was also obtained for the NP cores (Fig. 1c,d—black line, Table 1). Following the PMA coating and subsequent transfer to aqueous media, DCS measurements were performed on the coated NPs, resulting in a shift towards an apparent smaller diameters (left shift from the pristine uncoated particles) (Fig. 1c,d—red line). A broadening of the NP-protein corona peak was observed for the 5 Au-PMA particles only, indicating the presence of particles dimers or the presence of a NP population that absorbed corona proteins differently.

**Table 1:** Average NP diameter and standard deviation of the gold NPs prior to and after exposure with the biological fluid, measured by different characterisation methods. Particle diameters obtained from DCS measurements assumed a particle density of 19.3 g cm^-3^.

Particle diameter, d (nm)	5 Au	25 Au	50 Au
TEM core (nm)	4.8 ± 0.7	26.9 ± 3.4	52.0 ± 7.5
DCS core (nm)	4.5 ± 0.6	26.3 ± 4.0	51.4 ± 2.4
DCS Au-PMA (nm)	2.4 ± 0.2	22.6 ± 0.1	46.8 ± 0.2
DCS Au-PMA-C (nm)	2.3 ± 0.2	22.3 ± 0.1	43.1 ± 0.6

### Core-shell model calculations

**Scheme 2:**
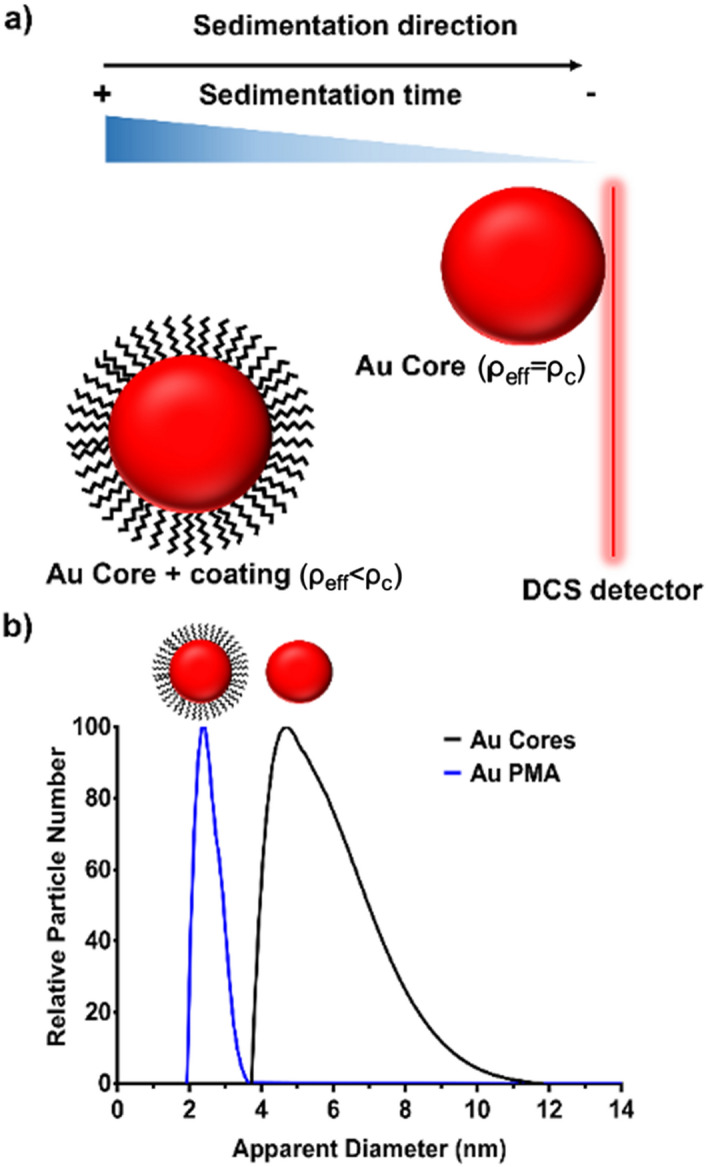
Schematic representation of the DCS measurements of bare and coated AuNPs. As described in Eqs. (1)–(3), the NP sedimentation time under a centrifugal force is directly correlated to their effective density. In the case of a bare AuNP, the effective density *ρ*^*eff*^ is close to the theoretical value (i.e. 19.3 g cm^−3^) while for a polymer coated AuNP, the effective density is affected (as described in the Eq. (3)) and also the sedimentation time. Scheme drawn with Adobe Photoshop.

The DCS measures the time it takes for the injected NPs to sediment and reach the light source (measuring point) whilst travelling through a linear density gradient fluid in a spinning hollow disc at a fixed rotational speed. During the measurement, the DCS software is using pre-set particle density values that are kept constant and equal to the one of the core materials for all measurements in accordance with the equation below:

1 $$t = \frac{C}{{(\rho_{eff } - \rho_{fl} ) d^{2} }},$$

where $\rho_{eff }$ is the density of the NP, $\rho_{fl}$ is the density of the fluid, and *C* a constant determined by a calibration process. Prior to each measurement, the value of the particle density ($\rho_{eff}$) is assumed. For a single component NP, e.g. a naked metallic core, its density is well known, and its size can be accurately determined, whereas, for composite nanomaterials, the density can often be estimated based on the known densities of the constituent materials (e.g. gold core has a known density of 19.3 g cm^−3^). However, when NPs are composed of several layers of different materials, $\rho_{eff }$ has to be estimated in order to calculate their overall diameter^12,13^. Typically, the addition of a ligand in the particle ligand shell lowers the overall particle density (*ρ*_*eff*_, in Eq. (2)), as the density of the ligand is often significantly lower than the one of the particle core material (Scheme 2).

A common way to estimate $\rho_{eff }$ is by the use of the core–shell model^33^ which assumes that the composite NP is composed of a core of known density and a number of different well defined layers, each of them with its own thickness and density. By calculating the total mass of the NP, which depends on the density and the thickness of each layer, and dividing it by the volume of the NP, we obtain the $\rho_{eff }$ given for the case of a core and a single layer by Eq. (2):

2 $$\rho_{eff } = \frac{{d_{c}^{3} \rho_{c} + [\left( {d_{c} + 2_{{s_{1} }} } \right)^{3} - d_{c}^{3} ]\rho_{{s_{1} }} }}{{\left( {d_{c} + 2s_{1} } \right)^{3} }},$$

while for the case of a core and two coating layers $\rho_{eff }$ is given by Eq. (3):

3 $$\rho_{eff } = \frac{{d_{c}^{3} \rho_{c} + [\left( {d_{c} + 2s_{1} } \right)^{3} - d_{c}^{3} \left] {\rho_{{s_{1} }} + } \right[\left( {d_{c} + 2s_{1} + 2s_{2} } \right)^{3} - \left( {d_{c} + 2s_{1} } \right)^{3} ]\rho_{{s_{2} }} }}{{\left( {d_{c} + 2s_{1} + 2s_{2} } \right)^{3} }},$$

where $d_{c}$ is the diameter of the core, *s*_*1*_ is the thickness of the first layer, *s*_*2*_ is the thickness of the second layer, and $\rho_{c}$, $\rho_{{s_{1} }}$ and $\rho_{{s_{2} }}$ are the densities of the core, the first layer, and the second layer, respectively (Scheme 1).

In practice, the size of a coated NP is determined with the following expression (Eq. (4)):

4 $$d_{NP}^{2} = \frac{{\left( {\rho_{c} - \rho_{fl} } \right)d_{DCS}^{2} }}{{\left( {\rho_{eff} - \rho_{fl} } \right)}},$$

where $d_{NP}$ is the total diameter of the NP which is $d_{NP}^{2}$ + 2s_1_ for the one-layer case and $d_{c}$ + 2s_1_ + 2s_2_ for the two-layer case, and $d_{DCS}$ is the NP size as measured with the DCS.

**Figure 2:**
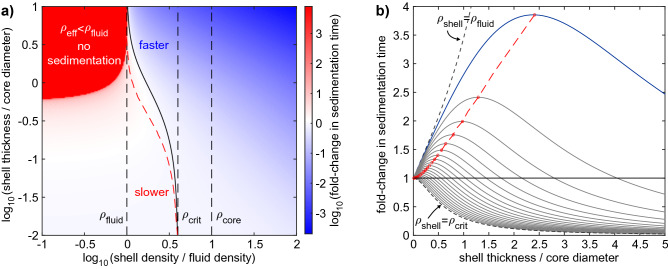
NP sedimentation time as a function of shell density and thickness. (**a**) Shown colour-coded is the fold-change in sedimentation time on a logarithmic scale (white to blue: faster, white to red: slower) relative to the sedimentation time of a bare NP without the shell. The sedimentation time is plotted double-logarithmically versus shell density and shell thickness. It was calculated using Eq. (1) by inserting the total (core plus shell) NP diameter, as well as the effective NP density $\rho_{eff}$ according to Eq. (2) (see “Methods” section). This plot uses normalised parameters in units $\rho_{fl} = 1$ (left vertical dashed line), $d_{c} = 1$, $t\left( {d_{s} = 0} \right) = 1$, and it assumes $\rho_{c} = 10$ (right vertical dashed line). The qualitative behaviour shown remains valid for all $\rho_{c} > \rho_{fl}$. Indicated are also the critical shell density $\rho_{crit}$ (middle vertical dashed line), as well as the maximum sedimentation time (red dashed line) for shell densities in the range $\rho_{fl} < \rho_{s} < \rho_{crit}$. (**b**) For different values of shell densities in the range $\rho_{fl} < \rho_{s}< \rho_{crit}$**,** the fold-change in sedimentation time increases and decreases as a function of the shell thickness. The red circles and dashed line represent the maximum sedimentation time for a certain shell density. Note that in theory two different shell thickness can give rise to the same fold change > 1, which is not a concern in the practical situation (blue curve) where the shell thickness never exceeds 2.5 times the core diameter. Image obtained with Matlab.

Since the shell not only changes the effective density of the NP but also its size, coupling these two effects will affect the particle sedimentation time in a non-trivial manner, as shown in Fig. 2. For a NP with a core density of $\rho_{c}$ sedimenting through a fluid of density $\rho_{fl}$, a shell density ($\rho_{s}$) larger than $\rho_{crit} > \left( {2\rho_{fl} + \rho_{c} } \right)/3$ will always lead to a faster sedimentation, whereas a density $\rho_{s} < \rho_{fl}$ will always result in longer sedimentation times, or no sedimentation at all, if the NP becomes effectively less dense than the medium itself (Fig. 2a, upper left corner). A shell with a density between these two limits can either slow down or accelerate sedimentation depending on its thickness. For thin shell coatings, the reduced effective NP density dominates and slows down the sedimentation, while for thicker shells, the larger size dominates and speeds up the sedimentation. At the boundary between these two regimens, particular combinations of shell thicknesses and densities do not change the NP sedimentation time at all (Fig. 2a, solid black curve). At a constant intermittent shell density $\rho_{c} < \rho_{s} < \rho_{crit}$, the sedimentation time increases with increasing the shell thickness up to a maximum (red dashed curve) before it decays again and becomes shorter than the sedimentation time of the bare NP (solid black curve, see also Fig. 2b). As an important consequence, increased sedimentation time is not related to a unique shell thickness for intermittent shell densities, but rather can, in theory, correspond to two different thicknesses. In the practical situation (see Scheme 1), the density of the polymer coating (shell thickness *s*_*1*_) and the density of the biomolecular corona (shell thickness *s*_*2*_) is much closer to the density of the surrounding medium than to the density of the core of gold NPs and the maximum sedimentation time would occur at a shell thickness of ca. 2.5 times the NP core diameter *d*_*c*_ (Fig. 2b, blue curve). For the smallest NP core diameter in this study (5 nm), this corresponds to a protein layer of 12.5 nm thickness. Since most proteins adsorb side-on and have a width in the range of 10 nm, thus less than 2.5 times the NP core diameter, an increase in shell thickness always results in an increase in sedimentation time. Therefore, as an output, the instrument will report a smaller apparent diameter as a result of the surface coating added.

Note, that also the 5 nm AuNP cores are coated with a thin organic ligand shell (dodecanethiol) before the polymer coating is added, and thus in principle the system might result with a core–shell system with different densities. However, when we considered the thin dodecanethiol shell a negligible change was observed and hence this it is not taken into account. Opposed to this, after the polymer coating addition, DCS measurements of 5 nm AuNPs coated with the PMA polymer of 2.4 ± 0.2 nm, resulting in a shift from the NP uncoated core of 2.1 nm (Fig. 1c,d, Table 1). Such shift confirms a change in the NP density and successful coating of the particles with the polymer, shown by the lack of any additional (side) peaks appearing, suggesting lack of any aggregation events.

We then evaluated whether the DCS could provide meaningful information on the particle’s colloidal stability after the exposure with a complex biological fluid, e.g. human plasma, in order to characterise the NP-biomolecular corona complexes (Au-PMA-C). For this purpose, we characterised NP-biomolecular corona complexes “in situ”, after incubation in blood plasma and without the removal of the excess of plasma (Fig. 1c,d, blue line) by centrifugation.

DCS analysis demonstrated that the resulting NP-biomolecular corona complexes were well dispersed in plasma, and strikingly, despite expecting a shift upon the corona formation, we observed an overlap of the two distributions for the 5 nm Au-PMA and 5 nm Au-PMA-C (Fig. 1c,d, red line). Similar results were observed for 25 nm AuNPs, where a shift towards smaller diameter (left shift from the NP uncoated core) occurred when the NPs were coated with the PMA polymer (Fig. 1g,h—black and blue lines) but no sedimentation shift was observed between 25 nm Au-PMA and 25 nm Au-PMA-C (Fig. 1g,h, blue and red lines). However, a different behaviour was observed for the 50 nm diameter AuNPs, where a shift was observed between the pristine and the PMA coated particles (Fig. 1k,l, black and blue lines) and a subsequent shift after the particles were incubated in situ in blood plasma (Fig. 1k,l, blue and red), confirming the presence of the biomolecular corona by means of the core shell model equations.

### NP-biomolecular corona validation

Based on these calculations, we found that the DCS is a reliable technique  for the quantification of the PMA shell thickness across different NP size ranges. However, the technique failed to detect a shift for the NPs-PMA-protein corona complexes for  f 5 and 25 nm sized particles of this particular density, highlighting a possible limitation of this technique.

This lack of shift in the measured diameter after sedimentation of 5 and 25 nm sized AuNPs could either implicate that the biomolecular corona forming around such small particles is rather a transient corona than the tightly and nearly irreversibly bound biomolecular layer i.e. “hard corona”, or that DCS may not be an ideal technique to resolve the NP-biomolecular corona complexes for small particles with a low density and the full characterisation would require different measuring techniques.

**Figure 3:**
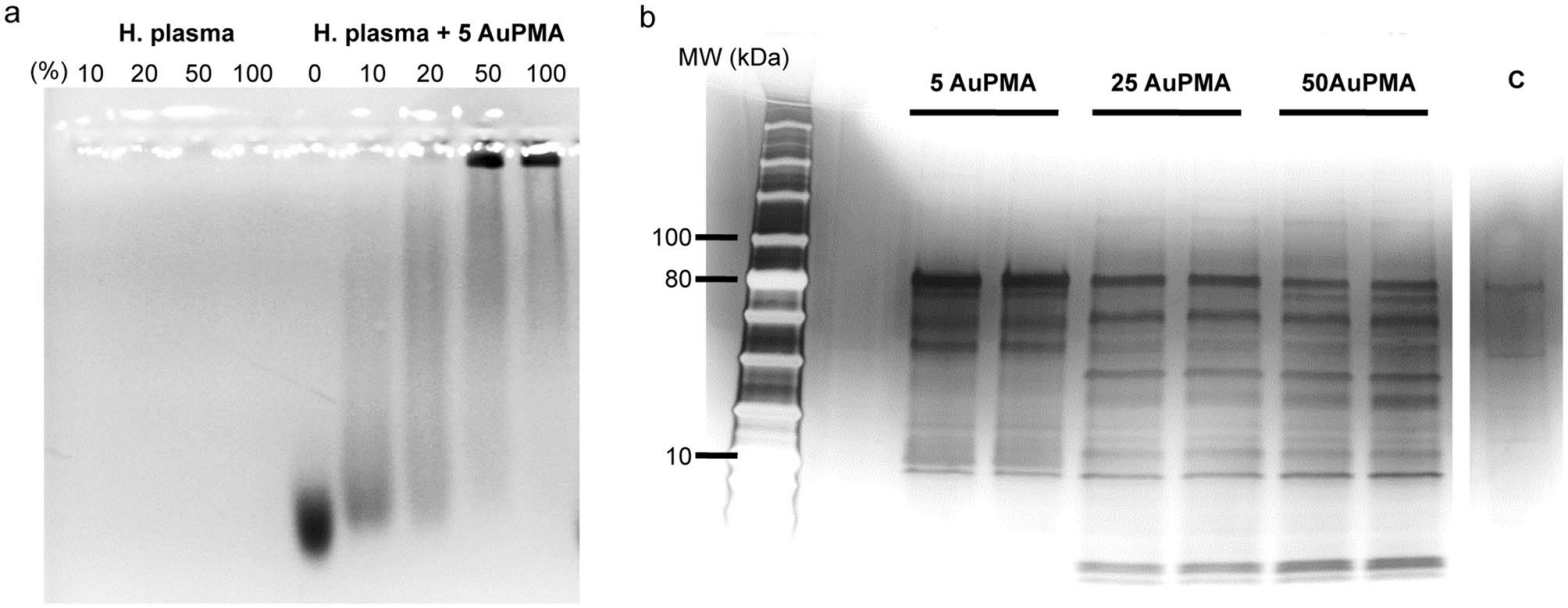
Electrophoresis analysis of the NP corona complexes. (**a**) Agarose gel electrophoresis assessed in a 2% (w/w) agarose gel of 5 nm AuNP before and after exposure to human plasma. The gel was visualized by a visible light scanner. (**b**) Silver stained SDS-PAGE gel showing the protein profile of the associated coronas for the different Au-PMA core sizes. The two lanes for each of the samples correspond to biological replicates, and the last one (C) represents the protein background (negative control). SDS-PAGE protein ladder corresponds to ColorPlus Prestained Protein Ladder, Broad Range (10–230 kDa) (New England Biolabs).

To address the presence of the biomolecular corona around 5 nm particles, we focused on the 5 nm Au-PMA particles and applied multiple methods to evaluate whether the strongly bound biomolecular corona is formed around these particles. For further characterisation, agarose gel electrophoresis (AGE) was applied, as the NPs generally migrate towards the anode as the travel through the gel pores, but any small changes in the particle surface, e.g. functionalisation with the organic ligands or indeed the biomolecular corona formation, might lead to a change in the electrophoretic mobility of the particles. Figure 3a shows that there is a clear electrophoretic mobility difference for the NPs after exposure to blood plasma, indicating that the formation of the corona influences the migration through the agarose gel, compared to the pristine ones.

The NPs with strongly bound biomolecular coronas, i.e. “hard corona” (HC) were isolated using centrifugation and we applied multiple washes to remove the unbound and loosely bound biomolecules from the corona^9^. However, due to their small size, we used an ultracentrifugation with a sucrose cushion to sediment the NPs-corona complexes. Briefly, after the incubation with human plasma, the NP-HC complexes were isolated by centrifugation using an ultracentrifuge for the 5 nm AuNP (55000 r.p.m., for 20 min) or a benchtop centrifuge for the 25 and 50 nm AuNP (5000 r.p.m., for 15 min) respectively, followed by 3 washes in phosphate buffered saline (PBS). The NP corona complexes were resuspended in PBS and characterised by Sodium dodecyl sulphate polyacrylamide gel electrophoresis (SDS-PAGE) to determine the presence and composition of the corona. To effectively separate leftover plasma proteins in the case of 5 nm AuNP-Corona, we applied a sucrose density cushion (40% w/v aqueous sucrose solution). This was performed in all 3 washing steps.

As shown in Fig. 3b, the PMA coated gold particles across all sizes display a strongly bound biomolecular corona as a result of distinct bands present in the gel. In particular, whilst the 25 and 50 nm Au-PMA NPs display a similar corona fingerprint, the smaller particles (5 nm Au-PMA) show a significant difference in the corona composition, suggesting that the surface curvature plays a significant role in the biomolecular corona formation of small particles. This hypothesis is in agreement with previous studies in which the biomolecular coronas of the PMA-coated AuNPs of different core sizes have been compared^19^.

Apart from the core size, the material of the particle core can significantly influence the biomolecular corona formation, e.g. due to the differences in density and inevitably different surface interactions attributable to differences in surface chemistry. However, for the polymer coated particles, this should not be the case, regardless of the core material. Hence, we explored the role of the particle core material in the biomolecular corona formation and we developed a series of inorganic particles with different core materials with the same core diameter, i.e. 5 nm namely, Ag, FePt NPs, and CdSn/ZnS quantum dots (QDs) all subsequently coated using the same PMA polymer^4^. As shown in Fig. S1, the PMA coating induced a change in the sedimentation with respect to the pristine particles for all materials tested. This is due to the change in the medium density as discussed previously, present in hybrid NPs composed out of an inorganic core and an organic polymer shell. Again, no additional shift occurred when these particles were exposed to the blood plasma, indicating that the sedimentation speed of the PMA and PMA-corona complexes was similar, in agreement with the 5 nm PMA coated AuNP . Similarly, to the 5 nm AuNP-PMA, also the PMA coated QDs resulted in a significant change in the electrophoretic mobility during the characterisation by agarose gel electrophoresis after exposure to blood plasma, indicating the presence of the biomolecular corona formation (Fig. S2). The biomolecular corona fingerprint was also characterised by  SDS-PAGE, where a specific corona fingerprint was observed depending on the biological fluid used during the incubation step (e.g. FBS, human plasma) (Fig. S3).

Overall, using the DCS as an only tool for characterisation of the NP protein coronas would lead to an incorrect conclusion that the PMA coating leads to a corona repulsion in the case of 5 and 25 nm sized particles. In fact, the gel electrophoresis data shown in this study demonstrate the biomolecular corona formation around these NPs.

### Application of the core–shell model

DCS has become a commonly used technique for the study of the particle dispersion properties in situ*,* evaluation of particle density, flotation properties, and even concentration estimation^13,33,38^. In particular, when combined with the use of a one- or two-layer density core–shell model, the coating thickness of different layers of chemical or biological relevance can be estimated. Here we have applied such knowledge to the characterisation of the Au-PMA NPs before and after corona formation. As previously mentioned, the NP diameter measured by the DCS is accurate only if the nanomaterial core density (*ρ*_*c*_) is known (see the Methods section for the definition of the core–shell). For the case of gold, which has a density of 19.3 g cm^−3^, by applying the core–shell model and solving Eq. (1), it is possible to calculate the true particle diameter. For sterically coated particles with polymers of lower density materials, a shift towards a smaller diameter is expected, as confirmed by the DCS measurements after the grafting process as an indication of the overall density change of the resulting coated NPs ^13,39^. In particular, for the AuNP shown in Fig. 1, after the PMA coating is applied, the density of the Au-PMA complex is reduced as the PMA layer has according to the applied core–shell model a density (*ρ*_*s1*_) of 0.9 g cm^−3^, which is significantly lower than the density of the gold core. By solving Eq. (4) for the net NP-core shell density (*ρ*_*eff*_), we confirmed that the PMA coating induced a significant drop of the particle overall density. The reduction in particle density is particularly pronounced in the case of Au-PMA NPs of 5 nm diameter, where we observe a decrease in the density of more than 90% compared to before coating. For the 25 and 50 nm NPs the reduction is less evident, close to 60% and 40%, respectively (Table 2). This is expected as in case the thickness of the PMA shell remains constant for all the core sizes, its relative contribution to the overall NP volume is the smaller the bigger the NP cores are.

**Table 2:** PMA and PMA corona shell thickness on AuNPs calculated using a core shell model on DCS measurements.

	5 Au	25 Au	50 Au
Au-PMA *ρ*^*eff*^[g cm^−3^]	1.5 ± 0.1	7.7 ± 0.1	10.9 ± 0.2
PMA layer [nm]*	5.3 ± 0.5	5.3 ± 0.1	5.9 ± 0.2
Au-PMA-Corona *ρ*_*eff*_ [g cm^−3^]	–	7.2 ± 0.1	7.0 ± 0.4
Corona layer [nm]*	–	0.5 ± 0.1	5.9 ± 0.9

*PMA and biomolecular corona thickness are calculated by applying the core–shell model as indicated in the Methods section.

Overall, based on the core–shell model (Eq. (3)), the measured polymer shell thickness of all NPs was of *ca.* 5.5 nm, indicate a similar coating regardless of the surface curvature. This is in good agreement with previous studies performed using different techniques^27^.

**Figure 4:**
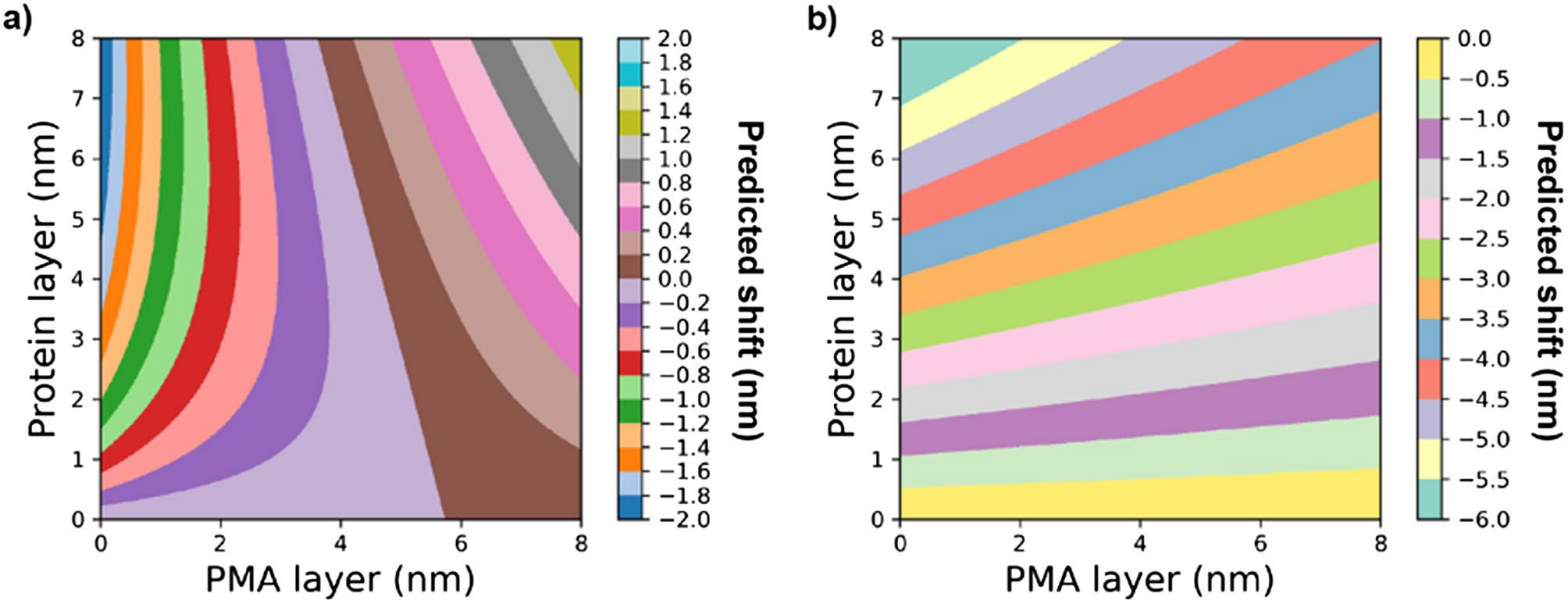
Predicted shift according to the core–shell model for different combinations PMA and corona thicknesses. (**a**) 5 nm and (**b**) 50 nm NP. Each coloured area corresponds to a predicted range of the shift.

Once the Au-PMA complex density and the thickness of the PMA layer were calculated, we subsequently estimated the thickness of the biomolecular coating and the density of the Au-PMA-biomolecular corona complex by using the two-layer model (Eq. (3) together with Eq. (4)). We assume a density of 1.15 g cm^−3^ for the protein layer as previously reported^13^. The results of these calculations are presented in Table 2. Firstly, we observed that for the 5 nm AuNP, the model could not be used to predict the biomolecular corona shell thickness due to an almost negligible DCS diameter shift (Table 1) combined with the sharp decrease of the effective density of the NP-PMA complex (Table 2). On the other hand, for the 25 and 50 nm sized AuNPs, the model does predict a size for the biomolecular corona, however for the 25 nm sized particles the obtained value was much smaller than expected^40^. To shed light on how the shift in the DCS diameter would predict a particular shell thickness for the PMA coating and the biomolecular corona complex, we calculated the expected shift for different combinations of PMA and protein corona thickness for the particle core sizes of 5 and 50 nm, using the core–shell model. The results are shown in Fig. 4, where each coloured band corresponds to a distinct range of the shift. In all cases, the density of the core, density of the PMA coating, and the biomolecular corona, were fixed to 19.3, 0.9, and 1.15 g cm^−3^, respectively.

For the 5 nm sized NPs, with a typical PMA coating of *ca.* 5 nm, and an average biomolecular corona of 5 nm thickness, a predicted shift in the measured diameter by the DCS would result between 0 and 0.2 nm because the opposing effects of the size increase and the reduced effective density nearly cancel each other out^13^. This is mainly due to the significant drop in the effective density of the resulting NP-polymer-corona complex, as the core which is usually larger than the outer layers contributes less to the total NP mass/density . It is also worth mentioning that smaller shifts than of 0.2 nm or less reach the limit of detection of the DCS analytical centrifuge, i.e., due to the instrument’s sensitivity, it would result indistinguishable by the standard deviation of the measurements. On the other hand, the same PMA layer and a biomolecular corona thickness of the 50 AuNP result in a theoretical change of the apparent diameter shift ranging between −3.5 nm and −3.0 nm, which is in agreement with the observed shift of -3.7 nm in our DCS measurements (Fig. 1).

Our core–shell model was applied for the estimation of the biomolecular layer on the surface of larger particles. In the case of the 50 nm Au-PMA the biomolecular corona layer of 5.7 nm was calculated, which is in agreement with previous estimates of the corona coatings by DCS, and with other methods^40^. Herein, applied techniques are better suitable for NPs with larger cores, which is in contrast to the diffusion-based techniques, e.g. FCS, where the biomolecular corona can be more efficiently detected around particles with smaller cores. This makes the DCS technique particularly attractive, it is complementary to other techniques and yields reliable results under conditions where other techniques would most definitely fail.

## Conclusion

In this article, we report a systematic study of the NP physico-chemical characterisation following the biomolecular corona formation applied to a custom-designed set of inorganic NPs where we varied the core size and material, and have applied  a surface coating with the PMA polymer, suitable for applications in nanomedicine. Our study shows that characterising small particle cores, i.e., 5 nm diameter with the DCS is not only experimentally challenging, owing to their small size and decreased overall particle density due to the PMA coating that requires long sedimentation times, but also the NP-corona complexes are not resolved by DCS as their density is not significantly different from the same NP without the corona, leading to a similar sedimentation time.

The designed workflow helps to rationally identify, characterise, and isolate small NP-biomolecular corona complexes by tuning and custom modifying existing methodologies. We have shown how the nature of the ligand can significantly affect the direction of the shift in the apparent diameter during the DCS measurements, which can be used for extrapolation of the biomolecular corona thickness. We have highlighted the unlikelihood of fully resolving the apparent diameter shift for very small NPs, where current models and approaches developed and readily applicable for larger NPs fail to confidently separate populations, even with high resolution instruments such as the DCS analytical centrifuge.

## Methods

### Nanoparticle synthesis and characterisation methods

Gold nanoparticles of 5 nm nominal core diameter were synthesised using the Brust-Schifrin method^35^**.** Subsequently, they were initially functionalised with the dodecanethiol and then transferred to aqueous phase by using the polymer coating technique coating the particles with the poly-(isobutylene-alt-maleic anhydride)-graft-dodecyl polymer (PMA).

Gold nanoparticles of 25 and 50 nm nominal core diameters were synthesised using the method reported by Bastús^41^, and subsequently transferred to chloroform and stabilised with dodecanethiol using the methodology reported by Soliman et al.^26^ and finally coated with the PMA polymer. The remaining nanoparticles (PMA coated FePt, Ag and CdSe/ZnS QDs) were synthetised according to a published protocol^4^.

All particles were characterised by UV–Vis absorption spectroscopy (Agilent 8453 spectrometer), inductively coupled plasma mass spectrometry (ICP-MS; Agilent 7700 series ICP-MS), TEM (Jeol 1400 plus), DLS (Nanosizer, Malvern), and in the case of water-soluble NPs via laser Doppler anemometry. The NP size distribution by TEM was obtained by imaging 279, 131 and 100 particles for 5 nm, 25 nm and 50 nm respectively and the images were processed by Image J software.

### Differential centrifugal sedimentation (DCS)

 The NP size distributions were analysed in dispersion using a CPS disc centrifuge DC24000 (CPS Instruments Inc.) using the appropriate density gradients depending on the nature of the sample. Sucrose solutions of 2–8% (w/v) with the density of 1.018 g cm^−3^, or 8–24% with the density of 1.064 g cm^−3^ (w/v) were used for the measurements of NPs with 5 nm core diameters and larger NPs, respectively and prepared in Milli-Q water or PBS for the measurements of the corona complexes, in situ, at pH 7.4. Sucrose solutions were prepared fresh and filled successively in nine consecutive steps into the disc, rotating at the speed set to 24000 r.p.m., starting with the highest density, according to the manufacturer’s guidance. Calibration was performed using a calibration standard, PVC 483 nm (Analytik Ltd.) before each analysis. Measurements were performed in a measurement range between 0 and 500 nm. The samples with the biomolecular corona were directly injected into the disc centrifuge without prefractionation or separation, to allow for the in situ analysis. Measurements of the hydrophobic NP cores dispersed in the organic solvent were performed using a toluene-based halocarbon density gradient composed by two solutions of 85% toluene + 5% Halocarbon 1.8 (w/w) and 95% toluene + 15% Halocarbon 1.8 (w/w) with resulting density of 0.915 g cm^−3^. In this case, diamond particles of 516 nm diameter were used as standard for measurements.

### Biomolecular corona preparation

The biomolecular corona preparation was carried out following a published method^12^. Briefly, the human plasma used for the biomolecular corona studies was obtained from the Irish blood transfusion service (IBTS) based in Saint James Hospital Dublin. The blood was collected from female and male healthy donors and processed by the IBTS. Formation of the biomolecular corona was performed for 1 h at 37 °C to allow the equilibration of the biomolecular corona, the ratio total surface area to protein was fixed in order to compare between NPs. The surface area of each NP applied was 2.5 × 10^15^ nm^2^ in a total of 500 μL, making a final surface area of 5 × 10^15^ nm^2^ mL^−1^.

### Agarose gel electrophoresis

Agarose gel electrophoresis was used to determine the interaction between the NPs and plasma proteins by measuring the NP electrophoretic mobility prior and after exposure to the biological media. The NP electrophoretic mobility was assessed using a 2% (w/w) low melting point agarose gel in Tris EDTA Buffer. A 50× Tris EDTA buffer stock was prepared by dissolving 24.2 g Trizma Base and 10 mL 0.5 M EDTA in 100 mL of Milli-Q water. Agarose was dissolved in 125 mL of 1× Tris EDTA buffer, after adjusting the pH to 8.5 with HCl, by heating in a microwave for 2 min and sonicated for 20 s.

NPs were incubated with the increasing plasma concentrations diluted in PBS buffer (10–100% blood plasma) and incubated for 1 h at 37 °C under shaking conditions. Subsequently, four parts of the sample were mixed with 1 part of Loading Buffer (36% v/v 5× Tris EDTA buffer, 50% glycerol, 0.01% w/v Bromophenol Blue) and 40 µL of this solution was loaded in each well. The electrophoresis was performed at 130 V in 1× Tris EDTA buffer for 90 min using a BioRad Agarose Electrophoretic System on ice to avoid overheating and melting of the gel.

### Isolation of the biomolecular coronas

Isolation of small NP-biomolecular corona complexes for the SDS-PAGE analysis were isolated using ultracentrifugation with a Beckman Coulter OptimaMax XP Ultracentrifuge equipped with a MLA-130 rotor, applying a cushion of 500 μL of 40% sucrose (w/v) solution to separate free proteins, the sample was dispensed on top and spun down for 30 min at 55000  r.p.m., the supernatant was removed, and the pellet was redispersed in 1× PBS. Three washes were performed in the same way. Larger NPs were spun down at 5000 r.p.m for 15 min and 3 washes in 1× PBS were performed prior to loading in 1D SDS-PAGE (10%) and run for 1 h at 130 V. The pellets were re-dispersed in PBS and mixed with 3× loading buffer (62.5 mM Tris–HCl pH 6.8, 2% (w/v) SDS, 10% glycerol, 0.04 M DTT and 0.01% (w/v) bromophenol blue), heated at 95 °C for 5 min and loaded for running. As molecular weight marker it was used the ColorPlus Prestained Protein Ladder, Broad Range (10–230 kDa) (New England Biolabs).

### Core-shell model analysis

The core-shell model analysis was carried out using an in-house written code where Eq. (4), along with Eq. (2), for a one-layer system, or Eq. (3), for the two-layer system, are numerically solved and Fig. 4 was obtained using the matplotlib library in Python. The numerical solution was needed as the equation to obtain the thickness is a polynomial of third order. It is worth noting that the densities of the core and each of the coating layers, PMA and biomolecule, are input parameters of the model. Also note that for a two-layer system, the thickness of the first layer is first calculated and the resulting value is then used in the calculation of the thickness of the second layer. These effective densities cannot be directly measured experimentally because their values are dependent on *d*_*c*_, the densities of all the components of the NP (core and the layers) and the thickness (s_1_ and s_2_) of the shells.

The following density values were used for the core shell model calculations: AgNPs (10.5 g cm^−3^), FePt NPs (4.0 g cm^−3^)^42^, CdSe/ZnS QDs (6.0 g cm^−3^), density of the PMA layer $\rho_{{s_{1} }}$ = 0.9 g cm^−3^ and density of the biomolecular corona coating ($\rho_{{s_{2} }}$) = 1.15 g cm^−3^^33^. The density of the fluids was 1.018 g cm^−3^ for the 5 nm NP measurements and 1.064 g cm^−3^ for the remaining measurements.

## Supplementary Information

Supplementary Information.
