# Supplementary material for: In depth characterisation of the biomolecular coronas of polymer coated inorganic nanoparticles with differential centrifugal sedimentation
Source: Sci Rep. 2021 Mar 19;11:6443. doi: 10.1038/s41598-021-84029-8 (PMC7979877; doi:10.1038/s41598-021-84029-8)
Supplement: Supplementary file 1 — Supplementary Information. [file 41598_2021_84029_MOESM1_ESM.docx]

**­­Supplementary information**

In depth Characterisation of the Biomolecular Coronas of Polymer Coated Inorganic Nanoparticles with Differential Centrifugal Sedimentation

**André Perez-Potti,^1,2^ Hender Lopez,^1,3^ Beatriz Pelaz,^4,5^ Abuelmagd M. Abdelmonem,^6,7,8^ Mahmoud G. Soliman,^6,9,10^ Ingmar Schoen,^11^ Philip M. Kelly,^1^ Kenneth A. Dawson,^1^ Wolfgang J. Parak,^6^ Zeljka Krpetic,*^1,12^ Marco P. Monopoli*^1,9^**

^1^ Centre for Bionano Interactions, University College Dublin, Dublin, Ireland

^2^ Center for Infectious Medicine, Department of Medicine Huddinge, Karolinska Institutet, Stockholm, Sweden

^3^ School of Physics and Optometric & Clinical Sciences, Technological University Dublin, City Campus, Kevin Street, Dublin 8, Ireland

^4^ Centro Singular de Investigación en Química Biolóxica e Materiais Moleculares (CiQUS), Universidade de Santiago de Compostela, 15782 Santiago, Spain

^5^ Departamento de Química Inorgánica, Grupo de Física de Coloides y Polímeros, Universidade de Santiago de Compostela,15782 Santiago, Spain

^6^ Fachbereich Physik, CHyN, University of Hamburg, Hamburg, Germany

^7^ Food Technology Research Institute, Agricultural Research Center, Cairo, Egypt

^8^ Institut für Physikalische Chemie und Elektrochemie, Leibniz Universität Hannover, Hannover, Germany

^9^ Chemistry Department, RCSI (Royal College of Surgeons in Ireland), 123 St Stephen Green, Dublin 2, Ireland

^10^ Physics Department, Faculty of Science, Al-Azhar University, Cairo, Egypt

^11^ School of Pharmacy and Biomolecular Sciences, RCSI (Royal College of Surgeons in Ireland), 123 St Stephen Green, Dublin 2, Ireland

^12^ Biomedical Research Centre, School of Science Engineering and Environment, University of Salford, M5 4WT Salford, United Kingdom

*Email: [z.krpetic@salford.ac.uk](mailto:z.krpetic@salford.ac.uk) and [marcomonopoli@rcsi.ie](mailto:marcomonopoli@rcsi.ie)


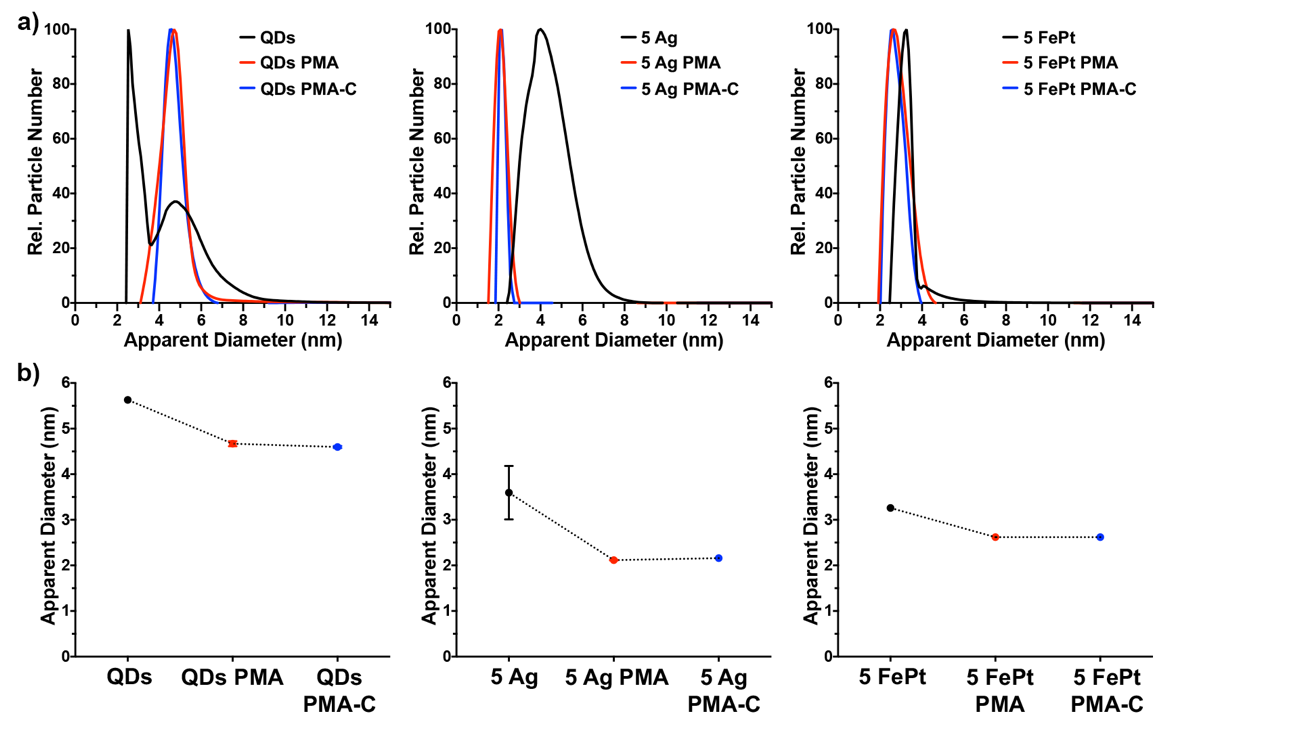


**Figure S1. DCS measurements of PMA coated ultra-small nanoparticles with different core composition.** Top panel shows representative DCS size distributions of the different samples for the three materials, namely, CdSe/ZnS QDs, AgNPs and FePtNPs. The bottom panel depicts the average maximum size for each of the dispersions for visualization of the shift.


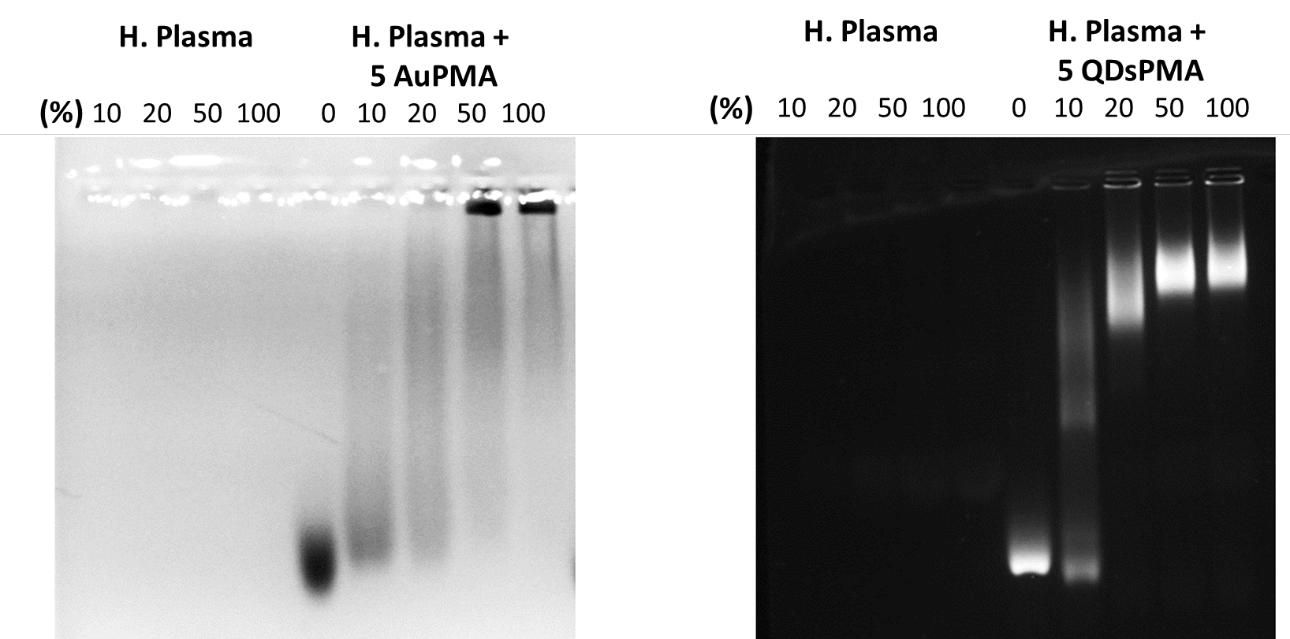


**Figure S2. Agarose gel electrophoresis for determination of level of interaction PMA USNPs-biomolecules.** Electrophoretic mobility is assessed in a 2% (w/w) agarose gel after incubation of the USNPs in increasing concentrations of human plasma. 5 nm QDs PMA USNPs were visualised by fluorescence scanner (488 nm excitation wavelength) in the presence and absence of biomolecules allowing to visualize the migration profile of the NPs in the presence and absence of biomolecular to understand the occurrence of interactions. Image taken with the Syngene G: BOX imaging system.


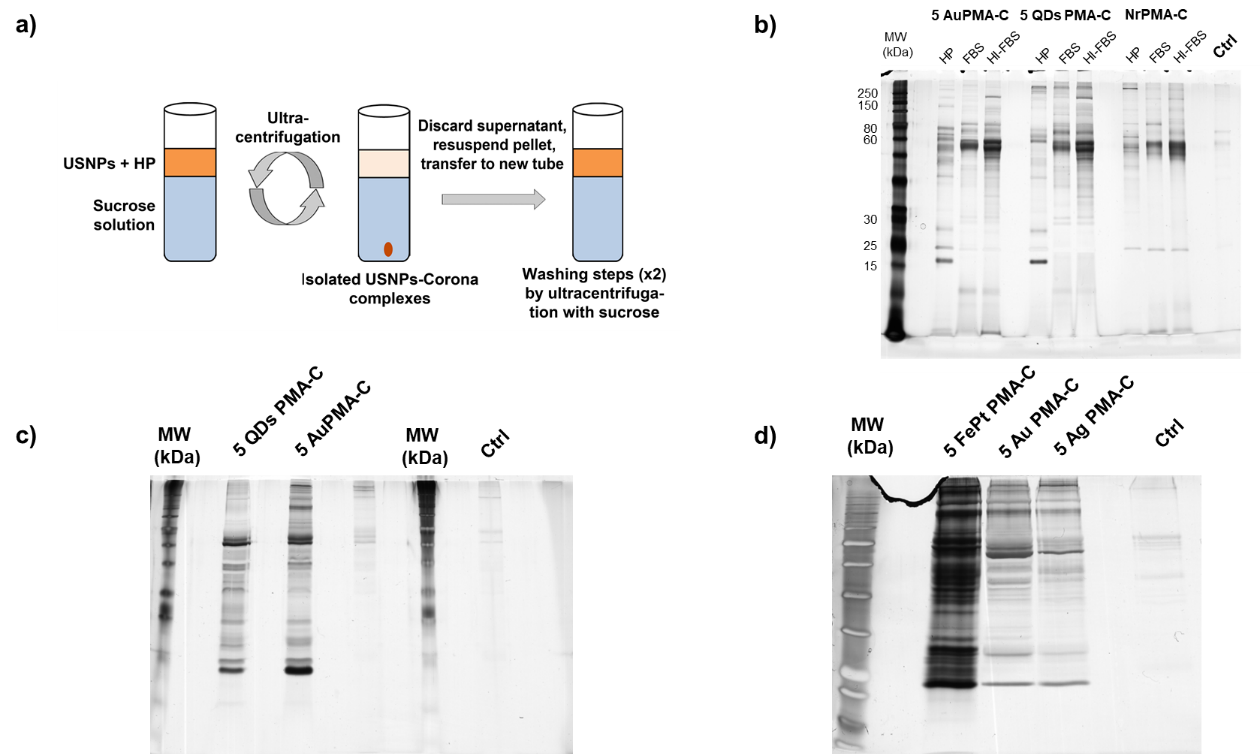


**Figure S3. Separation of NP-biomolecular corona complexes and analysis of associated coronas to PMA coated particles.** (a) Schematic representation of the isolation procedure of the NP using ultracentrifugation. (b) SDSPAGE of the biomolecular corona formed around. The isolated corona associated to the different NPs is shown in the different gels. The associated corona in different biological media (i.e. human plasma, HP; foetal bovine serum, FBS and heat inactivated foetal bovine serum, HI-FBS) was isolated for QDs and Au PMA coated NPs showing characteristic patterns, in addition to a larger Au NP tested for comparative purposes (b). Such procedure was repeated (b) for further prove the presence of a corona in comparison with background sample (Ctrl) and also done by another researcher to evaluate the procedure (d).
